# Understanding Frailty in Cardiac Rehabilitation: A Scoping Review of Prevalence, Measurement, Sex and Gender Considerations, and Barriers to Completion

**DOI:** 10.3390/jcm14155354

**Published:** 2025-07-29

**Authors:** Rachael P. Carson, Voldiana Lúcia Pozzebon Schneider, Emilia Main, Carolina Gonzaga Carvalho, Gabriela L. Melo Ghisi

**Affiliations:** 1 Faculty of Health, York University, Toronto, ON M3J 1P3, Canada; 2 Ciências da Saúde e do Esporte (CEFID), State University of Santa Catarina, Florianopolis 88080-350, Brazil; 3 Library & Information Services, Toronto Rehabilitation Institute, University Health Network, Toronto, ON M5G 2C4, Canada; 4 KITE Research Institute, Toronto Rehabilitation Institute, University Health Network, Toronto, ON M5G 2A2, Canada; 5 Division of Physical Medicine and Rehabilitation, Department of Medicine, Temerty Faculty of Medicine, University of Toronto, Toronto, ON M5S 1A1, Canada; 6 Department of Physical Therapy, Temerty Faculty of Medicine, University of Toronto, Toronto, ON M5S 1A1, Canada

**Keywords:** cardiac rehabilitation, frailty, barriers to care, sex differences, gender identity, health equity, secondary prevention, older adults, participation, access to care

## Abstract

**Background/Objectives**: Frailty is a multifactorial clinical syndrome characterized by diminished physiological reserves and increased vulnerability to stressors. It is increasingly recognized as a predictor of poor outcomes in cardiac rehabilitation (CR). However, how frailty is defined, assessed, and addressed across outpatient CR programmes remains unclear. This scoping review aimed to map the extent, range, and nature of research examining frailty in the context of outpatient CR, including how frailty is measured, its impact on CR participation and outcomes, and whether sex and gender considerations or participation barriers are reported. **Methods**: Following the PRISMA-ScR guidelines, we conducted a comprehensive search across six electronic databases (from inception to 15 May 2025). Eligible peer-reviewed studies included adult participants assessed for frailty using validated tools and enrolled in outpatient CR programmes. Two reviewers independently screened citations and extracted data. Results were synthesized descriptively and narratively across three domains: frailty assessment, sex and gender considerations, and barriers to CR participation. The protocol was registered with the Open Science Framework. **Results**: Thirty-nine studies met inclusion criteria, all conducted in the Americas, Western Pacific, or Europe. Frailty was assessed using 26 distinct tools, most commonly the Kihon Checklist, Fried’s Frailty Criteria, and Frailty Index. The median pre-CR frailty prevalence was 33.5%. Few studies (n = 15; 38.5%) re-assessed frailty post-CR. Sixteen studies reported sex or gender data, but none applied sex- or gender-based analysis (SGBA) frameworks. Only eight studies examined barriers to CR participation, identifying physical limitations, emotional distress, cognitive concerns, healthcare system-related factors, personal and social factors, and transportation as key barriers. **Conclusions**: The literature on frailty in CR remains fragmented, with heterogeneous assessment methods, limited global representation, and inconsistent attention to sex, gender, and participation barriers. Standardized frailty assessments and individualized CR programme adaptations are urgently needed to improve accessibility, adherence, and outcomes for frail individuals.

## 1. Introduction

Frailty is a multidimensional clinical syndrome characterized by reduced physiological reserve and resilience, resulting in increased vulnerability to stressors [1]. It typically manifests through the accumulation of health deficits over time and affects a substantial proportion of older adults [2], particularly those living with cardiovascular disease (CVD) [3]. Among this population, frailty prevalence ranges from 20% to 70% [1,3] and is associated with numerous adverse outcomes, including hospitalization, disability, falls, and mortality [3,4]. As life expectancy increases and the burden of CVD grows globally, addressing frailty has become a critical component of secondary prevention efforts [2].

Cardiac rehabilitation (CR) is an evidence-based, comprehensive model of secondary prevention for individuals living with CVD [5]. CR has demonstrated significant benefits, including improved functional capacity, reduced morbidity and mortality, and enhanced quality of life [6]. Importantly, these benefits directly counteract the impairments associated with frailty, positioning CR as a promising intervention to mitigate its impact in older adults with CVD [1,2]. However, despite these well-established benefits, CR remains markedly underutilized [7]—particularly among frail individuals, who face disproportionately low rates of referral, enrollment, adherence, and completion [2,8]. A recent systematic review highlighted that frail individuals are significantly underrepresented in CR programmes [2], pointing to the need for more inclusive and tailored models of care that reflect the complexities and heterogeneity of frailty.

Sex and gender are distinct but interrelated determinants of health. While sex refers to biological characteristics typically categorized as male or female, gender encompasses socially constructed roles, identities, and relationships [9]. Both influence health behaviours, access to care, symptom expression, and outcomes in CVD [10]. Research has consistently shown that women and gender-diverse individuals encounter greater barriers to CR participation and are less likely to complete programmes compared to men [11,12]. Furthermore, women with CVD are more likely to be frail and to experience worse outcomes than their male counterparts [13,14]. The intersection of frailty with sex and gender may further compound existing inequities in cardiovascular care [15], yet little is known about how this intersection is addressed in CR research, policy, or practice.

Given these critical gaps, a comprehensive understanding of how sex, gender, and frailty intersect to shape CR access, participation, and outcomes is urgently needed. This scoping review aimed to address this gap by mapping the existing evidence. Specifically, the objectives are: (a) to describe the characteristics of studies assessing frailty in the context of CR, including how frailty is defined and measured, and to examine the reported prevalence of frailty at CR entry and following CR completion; (b) to synthesize sex- and gender-related considerations in CR for individuals living with frailty; and (c) to identify barriers to adherence and completion of CR in this population.

## 2. Materials and Methods

Methods for this scoping review were based on the Preferred Reporting Items for Systematic reviews and Meta-Analyses extension for Scoping Reviews (PRISMA-ScR) Checklist [16]. The protocol was registered on Open Science Framework (OSF: https://osf.io/fpdcy/, accessed on 7 July 2025).

### 2.1. Eligibility Criteria

Eligible studies were peer-reviewed research publications that examined CR in relation to frailty. Studies were required to involve adult participants enrolled in Phase II outpatient CR programmes, defined as structured, multidisciplinary interventions that include an exercise component and are aimed at the secondary prevention of cardiovascular disease. To be included, studies had to assess frailty using a validated measurement tool, with frailty defined as a clinical syndrome of increased vulnerability due to diminished physiological and/or cognitive reserve [4]. Studies that included pre-frail or non-frail individuals were eligible if relevant comparisons were made. All primary study designs—quantitative, qualitative, and mixed methods—were considered. Reviews (systematic, scoping, or narrative) were excluded from analysis but screened to identify additional eligible primary studies. Studies were required to report outcomes related to frailty, such as prevalence, frailty scores, or associations between frailty and CR outcomes (e.g., adherence or completion). Studies that also addressed barriers to CR participation or incorporated sex- or gender-based analyses (SGBA) were considered of added value and prioritized in the synthesis. Only studies published in English were included; non-peer-reviewed literature, such as conference abstracts and editorials, was excluded.

### 2.2. Information Sources and Search Strategy

The following databases were searched from inception to 15 May 2025: Medline (Ovid MEDLINE(R) ALL), Embase (Embase Classic + Embase), CINAHL Ultimate (EBSCOhost), CENTRAL (EBM Reviews—Cochrane Central Register of Controlled Trials), Emcare (Ovid Emcare Nursing) and Web of Science. The search strategies were developed in collaboration with an Information Specialist (EM) based on the eligibility criteria related to frailty, CR, barriers to participation, and sex and gender considerations. Each database utilized relevant subject headings and free text terms to ensure retrieval of all relevant materials. Additionally, included studies’ references were reviewed by citation screening to identify any publications not included through database searching. The detailed search strategy is provided in Supplementary File S1.

### 2.3. Selection Process

Duplicate citations from various databases were eliminated using EndNote software (Version 21), and the unique citations were imported into Covidence. Two researchers (RPC and GLMG) independently screened all titles and abstracts identified by the search strategy for inclusion. To be included, abstracts had to identify frailty assessment and outpatient CR. The full texts of potentially eligible citations were subsequently assessed for eligibility (RPC and GLMG) based on the inclusion and exclusion criteria. Any disagreements were resolved by discussion between the two reviewers (RPC and GLMG).

### 2.4. Data Extraction and Synthesis

One researcher (RPC) independently extracted data on the following: population characteristics (including frailty level, sex, gender, and age), intervention components (including location, components, and duration), and barriers to CR participation or adherence. Results were synthesized in tabular form to provide an overview of the evidence and to identify gaps related to sex, gender, and frailty in CR research and practice using Microsoft Excel. Subsequently, the extracted data underwent independent review and validation by a second researcher (GLMG). Any disparities were resolved through consensus.

Data were synthesized using a combination of descriptive summary and narrative analysis approaches. Descriptive summaries were used to characterize the included studies. We then conducted a narrative synthesis of the data, organizing findings into three key domains: (a) frailty assessment methods and prevalence in CR; (b) incorporation of sex and gender considerations; and (c) reported barriers to CR adherence and completion among frail populations.

## 3. Results

### 3.1. Selection of Sources of Evidence

**Figure 1:**
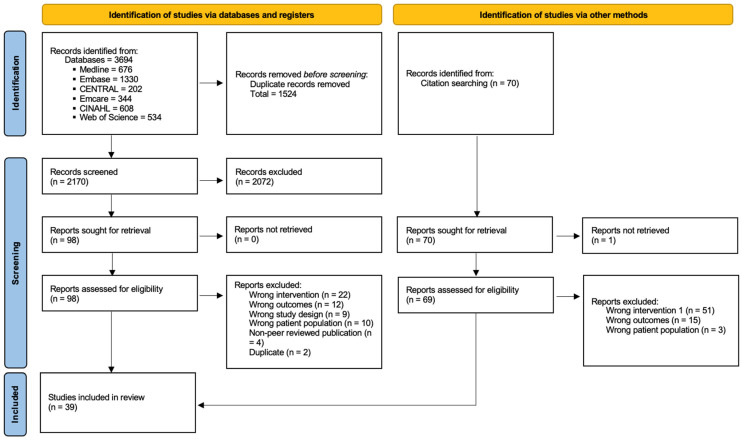
PRISMA flow diagram.

The initial database search yielded 3694 records. After excluding studies that did not meet eligibility criteria, 98 full-text articles were assessed for eligibility. Overall, 39 studies were included in this scoping review. Figure 1 presents the PRISMA flow diagram.

### 3.2. Study Characteristics

Table 1 summarizes the characteristics of the included studies. The first study was published in 2013 [17], and the most recent studies were published in 2024 [8,18,19]. Of the 39 included studies, all were quantitative: 13 (33.3%) retrospective cohort studies [18,20,21,22,23,24,25,26,27,28,29,30,31], 12 (30.8%) prospective cohort studies [17,32,33,34,35,36,37,38,39,40,41,42], 6 (15.4%) cross-sectional studies [19,43,44,45,46,47], 5 (12.8%) retrospective observational studies [8,48,49,50,51], and 3 (7.7%) randomized control trials [52,53,54]. Of these, 25 were full manuscripts [8,18,22,23,24,27,30,31,32,33,34,36,38,39,42,43,45,46,48,49,50,51,52,53,54], 12 were abstract-only publications [17,19,20,21,25,26,28,29,37,40,41,47], and 2 were research letters [35,44]. Study settings varied: 7 were multicentre studies [20,23,32,41,49,50,52] and 27 were conducted at a single centre [8,18,19,21,22,24,25,27,28,30,31,33,34,35,36,37,38,39,40,43,45,46,47,48,51,53,54]. Geographically, studies were conducted across 3/6 World Health Organization (WHO) [55] regions, including the Americas (n = 16), Western Pacific (n = 16), and Europe (n = 7). All studies were published in English.

Across the included studies, participant characteristics varied widely (Table 1). Sample sizes ranged from 27 to over 570,000 individuals, with the proportion of women ranging from 0.8% to 100%. Mean participant ages spanned from 53 to over 84 years, with several studies reporting stratified age means based on frailty status or intervention groups. Most participants were individuals living with CVD, including heart failure (HF), coronary artery disease (CAD), myocardial infarction (MI), valvular heart disease (VHD), and those recovering from cardiac surgery or procedures such as percutaneous coronary intervention (PCI), coronary artery bypass graft (CABG), or transcatheter aortic valve replacement (TAVI). Common comorbidities included hypertension, diabetes mellitus, dyslipidemia, atrial fibrillation, and chronic kidney disease. Some studies also reported psychosocial characteristics (e.g., depression, anxiety, education level, marital status), particularly in relation to frailty or rehabilitation engagement.

CR programme characteristics are described in Table 2. Twenty-two (56.4%) of programmes were centre-based [8,22,23,24,26,27,29,30,32,33,34,36,37,38,39,40,42,43,47,51,52,54], 4 (10.3%) used a hybrid model combining supervised and non-supervised components [18,48,49,50], and 2 (5.1%) were home-based [31,53]. Programme duration ranged from 3 weeks to 6 months, with the most common durations around 12 weeks (i.e., reported in 8 studies). Session frequency varied widely, from once weekly to daily sessions, with many programmes delivering exercise training 2 to 3 times per week combined with 1 education session weekly. The core components across most programmes included exercise training and patient education; several also incorporated lifestyle modifications, psychological or psychosocial support, medical evaluation, and nutrition guidance. One study (2.6%) indicated having a women’s only CR programme [25]. However, 5 studies [17,20,21,35,46] did not provide complete details about programme mode, duration, frequency, or components.

### 3.3. Frailty Assessment

Frailty was measured using 26 different assessment tools, described in Table 3. These tools assessed multiple domains such as physical function, exhaustion, grip strength, gait speed, and cognitive status, with frailty defined according to specific score thresholds unique to each instrument. The most commonly used frailty measures were: the Kihon Checklist (n = 6 studies) [38,43,45,46,47,53], the Fried’s Frailty Criteria (n = 6 studies) [17,29,31,50,52,54], the 25-Item Frailty Index (n = 5 studies) [8,22,39,40,51], and the 6-Minute Walk Distance (n = 4 studies) [26,27,37,41]. Most studies (n = 37; 94.9%) assessed frailty prior to the initiation of CR, with a median pre-CR frailty prevalence of 33.5% (interquartile range [IQR]: 25.8–39.8%; range: 10.6–100%). However, fewer studies (n = 15; 38.5%) conducted or reported post-CR frailty reassessment. The median post-CR frailty prevalence was 19.2% (IQR: 6.5–25.0%; range: 0–34%). Among those that assessed frailty after CR, improvements in frailty status were noted in 8 cohorts [18,21,29,33,37,42,49,54], suggesting potential benefits of CR on frailty reduction.

### 3.4. Sex and Gender Considerations

Of the 39 included studies, 16 (41.0%) incorporated some consideration of sex and/or gender. Among these, nine (56.3%) explicitly used the term sex [8,18,22,27,30,38,39,43,51], three studies (18.7%) used the term gender [32,33,34], one study (6.3%) used both terms interchangeably [49], and three studies (18.7%) used alternative descriptors without clarifying sex or gender terminology (e.g., “women” or “male/female”) [25,42,44]. No studies reported including non-binary or gender-diverse participants. Furthermore, no study adhered to equity-oriented research guidelines such as the SAGER (Sex and Gender Equity in Research) guidelines [57], which aim to promote the systematic consideration of sex and gender across all stages of research—from study design and data analysis to interpretation and reporting. Nor did any use sex and gender-based analysis (SGBA) frameworks or tools.

Among the 16 studies reporting sex or gender, 13 (81.2%) disaggregated frailty data by sex/gender or included sex/gender as a covariate [8,18,25,27,30,32,38,39,42,43,44,49,51]. The most commonly reported comparisons involved prevalence of frailty across sexes or genders, subgroup analyses, and multivariable models including sex/gender. Among the 9 studies that reported the proportion of women with frailty [25,27,30,32,38,42,43,48,49], the median prevalence was 38.0% (IQR: 18.6%; range: 0% to 58%). Five studies stratified frailty prevalence by sex, consistently showing higher prevalence among women compared to men across various CR settings [8,18,34,39,44]. In studies where significant sex differences were reported, women were found to have higher frailty levels both pre- and post-CR, with one study explicitly associating female sex with lower CR goal achievement [8]. Only two studies conducted interaction or sensitivity analyses to examine sex- or gender-specific effects of CR on frailty outcomes, and both reported no significant interactions [22,34].

### 3.5. Impact of Frailty and Barriers to CR Participation in Patients with Frailty

Seventeen (43.6%) studies reported data on frailty and CR participation [8,18,20,22,23,24,28,30,32,39,40,42,44,48,49,50,51]. Overall, frail patients were less likely to attend or complete CR programmes compared to non-frail patients. CR attendance among frail individuals ranged from approximately 9.3% to 13.7%, whereas attendance rates in non-frail groups were consistently higher, reaching up to 30%. Increasing frailty was associated with decreased CR use, with one study [20] showing a decline in participation from 48.3% in the least frail quartile to 20.6% in the most frail (*p* < 0.001), and adjusted odds ratios indicating significantly lower odds of CR participation with higher frailty scores (OR 0.61, *p* < 0.001). Frailty scores were generally lower among CR participants compared to non-participants (e.g., 0.28 vs. 0.33) [23], and those engaged in hospital- or home-based rehabilitation had significantly lower frailty scores than those not participating in any rehabilitation (*p* < 0.0001) [18]. Although frailty levels did not consistently predict achieving specific CR goals when categorized, every 1% increase in the frailty index at admission was associated with a 1.2% reduction in the odds of reaching a CR goal [8]. CR completion rates were lower among the frailest groups, with non-completers showing higher frailty scores and more frailty criteria [8,22,24,48]. However, improvements in frailty status were observed following CR in some studies, with up to 87% of frail patients shifting to non-frail status after a 3-month outpatient programme [42]. Overall, these findings highlight that while frailty is linked to lower CR participation and completion, engagement in CR can contribute to improvements in frailty status.

Eight (20.5%) studies reported barriers that hinder CR participation among patients with frailty [22,37,38,43,44,49,50,54]; Table 4. A recurring barrier was the interplay between frailty and physical limitations, including reduced strength, balance, and functional capacity. These limitations often necessitate tailored modifications to CR programmes to address the specific needs of frail patients, such as additional focus on balance training, strength-building, and cognitive support [37,44]. Mental health and emotional distress emerged as a barrier, with studies linking frailty to increased risk of anxiety, depression, and lower scores on the role-emotional domain of the 36 Item Short Form Survey (SF-36) [22,38,43]. Lack of transportation was identified as the most frequently reported logistical barrier, accounting for 41% of nonparticipation cases overall and disproportionately affecting older adults [44]. As age increased, so did the prevalence of transportation issues (rising from 25% in ages 65–74 to 49% in those aged ≥85, *p* < 0.01). Conversely, lack of motivation was reported less frequently among the oldest groups, suggesting a shifting profile of barriers with ageing [44]. Healthcare system-related factors were identified including conflicting medical appointments resulting in 36% adherence for CR participants [50]. Personal and social factos were identified including personal commitments, lack of social support and caregiver responsibilities [50]. Some studies also noted that frail individuals were significantly less likely to meet baseline physical activity (PA) guidelines, suggesting underlying deconditioning and low PA habits as contributing factors to low CR engagement [49]. Although cognitive impairment was not directly quantified in the included studies, it was mentioned as a concern, reinforcing the need for CR programme adjustments to support executive functioning and decision-making among frail participants [37].

## 4. Discussion

This scoping review provides a comprehensive synthesis of current evidence on the intersection of frailty and CR, with a particular focus on barriers to participation and sex- and gender-related considerations. A total of 39 studies were included, spanning diverse populations, CR settings, and frailty assessment methods. Despite consistent evidence that frailty is associated with reduced CR referral, attendance, and completion [2,51,58], engagement in CR remains notably low among frail individuals. Importantly, approximately 1 in 3 individuals entering CR were living with frailty, with reported prevalence ranging from 10.6% to 100% (median: 33.5%). This highlights both the substantial clinical burden of frailty in CR populations and the considerable heterogeneity in how frailty is measured across studies. While improvements in frailty status were observed post-CR in some studies, frailty remained a significant predictor of lower programme adherence and goal achievement. Notably, sex- and gender-based analyses were limited; only 16 studies incorporated any sex or gender considerations, and none included gender-diverse populations or applied equity-oriented research frameworks. Furthermore, barriers to CR among frail patients were multifaceted, including physical and cognitive limitations, emotional distress, transportation challenges, and low baseline physical activity. These findings underscore the urgent need to develop inclusive, tailored CR models that address both frailty-specific barriers and sex/gender-related inequities in access and outcomes.

Standardizing frailty definitions and assessments has been proposed as a necessary step to accurately characterize and diagnose frailty in CR populations [2], particularly given that frailty is not synonymous with older age and requires formal assessment [59,60]. However, findings from this scoping review demonstrate that this goal remains unmet. The included studies revealed wide variability in the tools used to assess frailty, ranging from validated instruments like the Fried phenotype [61] and Clinical Frailty Scale [62] to less clearly defined measures. Some studies lacked explicit definitions altogether or relied on proxy terms, undermining comparability and consistency. Additionally, not all included studies were full manuscripts, further limiting transparency in how frailty was conceptualized and measured. This heterogeneity in definitions and methodologies makes it challenging to accurately estimate frailty prevalence, evaluate outcomes, and design tailored interventions for this population. To advance the field, a consensus on a standardized, validated frailty assessment tool and operational definition is urgently needed for both research and clinical CR settings [60]. Routine and consistent use of such tools could enhance diagnostic accuracy, support individualized programme adaptations, and ultimately improve CR access, adherence, and outcomes for patients living with frailty [63,64].

Despite the recognized importance of applying a sex- and gender-based analysis in cardiovascular research [65], our scoping review found limited integration of SGBA in studies examining frailty in the context of CR. While some studies reported sex-stratified data, few explored gender-related factors such as caregiving roles [66], social support [67], or access to care [68]—elements that may significantly influence CR participation and outcomes in individuals living with frailty. Moreover, none of the included studies incorporated a comprehensive SGBA framework or examined how sex and gender intersect with frailty to impact CR adherence or completion. Notably, of the 34 studies that reported the percentage of women in CR programmes, only five included a majority female sample (i.e., >50% women), underscoring the persistently low participation of women in CR. This represents a critical gap, as frailty may present differently and be experienced uniquely across sex and gender identities. Furthermore, the predominance of binary sex/gender categorization in the literature fails to capture the experiences of non-binary and transgender individuals, highlighting the need for future research to adopt multilevel gender analyses that reflect this diversity. Future research must move beyond binary reporting and actively integrate SGBA methodologies to better understand the complex interplay of biological and sociocultural factors in this population. To support this shift, we recommend the use of the SAGER guidelines [57] to systematically incorporate sex and gender considerations throughout the research process. Doing so would support the development of more equitable and responsive CR programmes tailored to the diverse needs of individuals living with frailty.

Only a limited number of studies explicitly addressed barriers to CR participation among patients with frailty, revealing an important research gap. The reported barriers include physical limitations such as reduced strength, balance, and functional capacity; psychological challenges like anxiety, depression, and low emotional functioning; logistical issues, most notably lack of transportation; and cognitive concerns related to executive functioning and decision-making. While many of these barriers overlap with those commonly reported in the general CR population [11,69], frail patients face unique challenges in severity and complexity, such as more pronounced physical deconditioning and greater cognitive vulnerability. Collectively, these multifactorial barriers—spanning physical, emotional, logistical, and cognitive domains—highlight the urgent need for CR programmes to move beyond a one-size-fits-all approach [70]. Instead, comprehensive and individualized adaptations are essential, including enhanced physical training tailored to frailty [71,72], integrated mental health support [73], flexible scheduling or transportation solutions [74], and cognitive accommodations [75]. Such tailored strategies are critical to improving accessibility, sustaining adherence, and ultimately optimizing clinical outcomes for this especially vulnerable group.

### 4.1. Limitations

This scoping review has several limitations that should be considered when interpreting the findings. Although a total of 39 studies were included—reflecting a growing interest in the intersection of frailty and CR—considerable heterogeneity in frailty definitions, assessment tools, and outcome measures limited the comparability of results across studies. Furthermore, key variables such as SGBA and barriers to CR participation were inconsistently reported, making it difficult to draw comprehensive conclusions. The geographical distribution of studies was also limited, with all included research originating from only three WHO regions (the Americas, Europe, and Western Pacific), which restricts the generalizability of findings to other global contexts. There is a clear need for future research in underrepresented regions such as Africa, South-East Asia, and the Eastern Mediterranean to ensure a more inclusive and globally relevant evidence base. Additionally, a portion of the included records (35.9%) were abstracts or research letters, which often provide limited methodological detail. The lack of reporting on key CR programme characteristics in many studies—as reflected in Table 2 for variables including location, modality, duration, frequency, and components—is notable, highlighting a broader issue of incomplete reporting in primary studies. This gap underscores the need for greater transparency and standardization in describing CR programme characteristics in future publications. Lastly, consistent with scoping review methodology, we did not conduct a formal quality appraisal of the included studies, which limits the ability to assess the overall strength and reliability of the evidence base. Future studies should aim to standardize frailty assessments, consistently report SGBA and barriers, and include diverse populations to better inform equitable and effective CR interventions for frail individuals. Additionally, there is a critical need for more studies assessing frailty after completion of CR programmes to better understand the longitudinal impact of CR on frailty status.

### 4.2. Practice Implications

The findings of this scoping review have several practical implications for the design and delivery of CR programmes for individuals living with frailty. The ageing patient population and increase in frailty prevalence indicate an urgent need to improve CR practices. First, the heterogeneity in frailty definitions and assessment tools highlights the urgent need for standardized screening and classification methods to better identify and tailor interventions for this population. Based on our findings, we were unable to determine the most appropriate tools or approaches for frailty assessment in CR settings, which underscores a critical gap that should form the basis of future research. Clinicians and programme developers should consider integrating targeted adaptations into CR programmes—such as strength and balance training, cognitive support, and mental health services—to address the multifactorial barriers identified, including physical limitations, emotional distress, and cognitive concerns. Additionally, logistical challenges like transportation should prompt greater implementation of flexible delivery models, including home-based and hybrid CR options. These challenges further highlight the need for system-level changes to prevent and manage frailty in high-risk patient populations. Given the underreporting of sex, gender, and sociodemographic data, there is also a need to improve equity-informed data collection and programme planning to ensure interventions are responsive to diverse needs. Moreover, our findings can inform frailty-specific CR guidelines by emphasizing the importance of standardized screening, tailored programme adaptations, and equitable access. Healthcare policies that support flexible, inclusive, and accessible CR models are essential to effectively address the complex needs of frail patients. Ultimately, a shift toward personalized, inclusive, and accessible CR models is essential to improve participation, adherence, and health outcomes among frail patients.

## 5. Conclusions

This scoping review highlights the growing yet still limited body of literature examining frailty within the context of CR. While evidence suggests that individuals living with frailty can benefit from CR, significant gaps remain in how frailty is defined, assessed, and addressed across programmes. Barriers to participation span physical, psychological, cognitive, and logistical domains, underscoring the need for individualized and adaptable CR models. The lack of global representation and inconsistent reporting of sex, gender, and other equity-related variables further limit the applicability of current evidence. Future research should prioritize standardized frailty assessments, equity-informed approaches, and the inclusion of diverse populations to ensure CR programmes are accessible, inclusive, and effective for people living with frailty. Addressing these gaps is essential to optimize CR outcomes and promote health equity in this vulnerable population.

## Figures and Tables

**Table 1:** Characteristics of included studies and participants (N = 39).

First Author Year Country	Aim	Study Design Setting	Participants	Total N, % Women Age: Mean ± SD (Range) or Median (Range)	Characteristics of Participants ^‡^
Adachi [32] 2023 Japan	To examine the effects of CR on the 2-year prognosis of patients with HF, according to their frailty status.	Prospective cohort study Multicentre (number of centres NR)	Patients hospitalized for HF and capable of walking at discharge	N = 2697, 39.6% women Overall: 76.0 (67–83) years Frailty CR group: 78.0 ± NR (74–83) years	Atrial fibrillation (35.1%), diabetes mellitus (34.8%) at admission
Baldasseroni [33] 2023 Italy	To examine the effect of standardized CR after ACS on physical frailty.	Prospective cohort study 1 centre	CR participants with ACS	N = 100, 20.0% women 80.8 ± 0.5 (75–94) years	CABG (17.0%), current smoking (14.0%), diabetes mellitus (20.0%), dyslipidemia (56.0%), hypertension (78.0%), NSTEMI (36.0%), STEMI (31.0%), valvular surgery (16.0%) at admission
Bauer [20] 2023 USA	To evaluate the relationship between preprocedural frailty, CR use, and one-year mortality.	Retrospective cohort study Multicentre (number of centres NR)	Patients who underwent inpatient percutaneous or surgical revascularization or aortic valve replacement	N = 570,851, % women: NR Age: NR	NR
Bencivenga [34] 2022 Italy	To determine the relationship between frailty and CR outcomes in hospitalized older adults.	Prospective cohort study 1 centre	Patients referring to CR after HF exacerbation, IHD, VHD, cardio-aortic surgery, and other CVD	N = 559, 30.8% women Overall: 72.0 (69–76) years Frailty group: 73.0 (69–77) years	HF (24.7%), IHD (41.5%), VHD (29.7%), Other CVD (4.1%) at admission
Dinesh [18] 2024 Australia	To identify whether patients with advanced heart disease participating in a hospital-based rehabilitation programme or a home-based structured exercise program had lower frailty scores compared with non-rehabilitation participants.	Retrospective cohort study 1 centre	Advanced heart disease (DCM, IHD, valvular, congenital, HCM RCM, other)	N = 124, 18% women Overall for heart disease group: 53.0 ± 12.0 (range NR) years Frailty group: 55.0 ± 10.0 (range NR) years Pre-frail group: 53.0 ± 13.0 (range NR) years Robust group: 54.0 ± 12.0 (range NR) years	CHD (5.6%), DCM (52.4%), HCM (4.0%), IHD (25.0%), RCM (3.2%), VHD (6.5%), other CVD (3.2%) at admission
Eichler [35] 2020 Germany	To investigate the feasibility and safety of functional and nutritional assessments in patients after PCI in CR.	Prospective cohort study 1 centre	Patients over or equal to 75 years of age after TAVI, AVI or PCI	N = 124, 47.6% women 81.8 ± 3.5 (range NR) years	Arrhythmia (10.5%), diabetes mellitus (32.3%), dyslipidemia (39.5%), hypertension (82.3%), infections (12.1%) musculoskeletal diseases (20.2%), renal insufficiency (21.8%) at admission
Fonteles Ritt [21] 2021 Brazil	To evaluate the association of the CR programme with frailty indicators in elderly patients with heart disease referred to a CR program.	Retrospective cohort study 1 centre	Patients over 65 years old referred to CR	N = 51, 35.0% women 75.0 ± 6.0 (range NR) years	CAD (77.0%), HF (50.0%), diabetes mellitus (41.0%), hypertension (67.0%), dyslipidemia (80.0%) at admission
Francesco-Cacciatore [17] 2013 Italy	To verify the prevalence of frailty and its predictive role on functional recovery after CR in elderly patients with CVD.	Prospective cohort study NR	Patients older than 65 years with CVD after a major cardiovascular acute event	N = 350, % women NR Age: NR	NR
German-Sallo [19] 2024 Hungary	To assess frailty in elderly patients, as part of a comprehensive CR programme and to identify the main components contributing to loss of self–care.	Cross-sectional study 1 centre	Patients aged 60 years or older admitted to CR	N = 92, 53.3% women 72.4 ± 7.4 (range NR) years	Atrial fibrillation (57.1%), CKD (46.5%), osteoarticular diseases (39%), stroke (26%) at admission
Gilbert [52] 2023 USA	To evaluate whether the effects of physical rehabilitation interventions vary across racial groups in ADHF.	Randomized control trial Multicentre (7 hospitals; 4 academic, 3 community-based)	Patients aged 60 years or older hospitalized for ≥24 h for ADHF, including both HF with preserved EF and HF with reduced EF	N = 349, 51.3% women 72.5 ± 3.0 (range NR) years	Hypertension (92.0%), atrial fibrillation (50.4%), hyperlipidemia (66.2%) at admission
Hashimoto [36] 2022 Japan	To examine whether adding robotic balance exercises to CR improved the balance ability of older adults with CVD.	Prospective cohort study 1 centre	Older adults who had been hospitalized for worsening CVD	N = 52, 46.2% women 76.9 ± 6.8 (65–95) years	Diabetes mellitus (15.4%), dyslipidemia (32.4%), and tobacco users (1.9%) at admission
Henderson [37] 2017 USA	To evaluate if CR may benefit frail patients by enabling functional gains that may even exceed relative improvements among non-frail patients.	Prospective cohort study 1 centre	Patients with CVD of diverse etiologies (CAD, HF, VHD)	N = 60, % women NR 68.0 (45–81) years	NR
Hillier [22] 2023 Canada	To determine which patient characteristics and age-related health deficits are important in patients completing CR.	Retrospective cohort study 1 centre	Patients who experienced acute CVD, including CAD, MI, PCI, CABG, valve surgery, HF, or a combination of other diagnoses, with few referrals (e.g., arrhythmia, heart transplant)	N = 4004, 25.5% women CR group: 62.4 ± 10.7 (range NR) years	CAD (27.0%), PCI (15.0%), cardiac surgery (20.0%), HF (7.0%), MI (28.0%), other (4.0%) at admission
Honzawa [38] 2022 Japan	To investigate the effects of phase II CR on physical function and anxiety levels based on the prevalence of frailty.	Prospective cohort study 1 centre	Patients who participated in early-phase II CR	N = 137, 29.2% women Overall: 66.4 ± 2.3 (range NR) years Frail: 69.0 ± 13.2 (range NR) years	Hypertension (67.9%), dyslipidemia (52.6%), diabetes mellitus (21.9%), current smoker (11.7%), open heart surgery (47.4%), HF (24.8%) at admission
Honzawa [43] 2020 Japan	To retrospectively examine the relationship between KCL score and anxiety levels in elderly patients undergoing early phase II CR.	Cross-sectional study 1 centre	Patients who participated in early-phase II CR	N = 255, 33.3% women Overall: 74.9 ± 5.8 (range NR) years Frail: 75.5 ± 5.8 (range NR) years	Hypertension (69.8%), dyslipidemia (47.1%), diabetes mellitus (31.4%), current smoker (14.1%) CVD diagnoses: IHD (52.9%), open heart surgery (60.4%), HF (22.0%) at admission
Iritani [44] 2023 Japan	To determine the reasons for non-participation of HF patients in CR and whether frailty impacted these reasons.	Cross-sectional study NR	Patients aged 65 years or older who were hospitalized for HF and were ambulatory at the time of discharge	N = 1993, 43.0% women 78.0 (73–84) years	NR
Kamiya [23] 2020 Japan	To estimate the impact of CR on prognosis in patients with HF.	Retrospective cohort study Multicentre (15 hospitals)	Patients hospitalized for HF	N = 3277, 41.1% women 74.9 ± 14.9 (range NR) years	Atrial fibrillation (30.0%), diabetes mellitus (40.0%), hypertension (69.0%) at admission
Kehler [39] 2020 Canada	To provide a comprehensive evaluation of frailty changes at CR completion in relation to admission frailty levels.	Prospective cohort study 1 centre	Patients enrolled in CR with CAD, MI, PCI, CABG, HF, or a combination of “other” diagnoses with low referral rates (eg, arrhythmia, heart transplant)	N = 3756, 15.3% women Overall: 62.6 ± 10.7 (range NR) years >0.50 FI (most frail): 62.0 ± (9.5) (range NR) years	CAD (27.5%), PCI (14.9%), MI (27.8%), cardiac surgery (19.3%), HF (6.5%), other (3.9%) at admission
Kimber [24] 2018 Canada	To determine the impact of pre-operative frailty on CR completion rates.	Retrospective cohort study 1 centre	Cardiac surgery patients undergoing either elective or urgent CABG and/or valve procedures	N = 114, 36.8% women Overall: 71.0 (66–78) years CR non-completers: 71.5 (66.3–78) years CR completers: 70.5 (66–72) years	MI (29.8%), HF (49.1%), diabetes mellitus (25.4%), CRF (3.5%), COPD (11.4%), depression (11.4%) at admission
Kunimoto [45] 2019 Japan	To evaluate the relationship between the Kihon Checklist and the clinical parameters in patients who participated in CR.	Cross-sectional study 1 centre	Patients with CVD (ACS, after open heart surgery or TAVI, HF, major vessel disease and PAD) who participated in phase II CR	N = 845, 30.8% women Overall: 70.6 ± 2.1 years Frailty: 73.0 ± 8.5 (range NR) years	MI (11.7%), PCI (16.7%), HF (24.1%), diabetes mellitus (34.6%), hypertension (64.1%), dyslipidemia (49.8%), current smoker (8.9%) at admission
Landry [25] 2018 Canada	To describe the prevalence of functional exercise testing modality in patients previously classified as “moderately frail” using the Frailty Index Scale in CR patients.	Retrospective cohort study 1 centre	Women living with CVD and participating in CR	N = 800, 100% women Moderate frailty: 65.8 ± 10.2 (44–92) years	NR
Lutz [26] 2019 USA	To examine if CR benefits frail adults as much as pre-frail and non-frail.	Retrospective cohort study NR	CVD patients who completed a phase II CR programme	N = 163, % women NR Age: NR	NR
Lutz [27] 2020 USA	To study changes in physical function among frail, intermediate-frail, and nonfrail older adults CR patients enrolled in a phase II CR programme.	Retrospective cohort study 1 centre	Patients with a diagnosis of HF, cardiac surgery, implantable cardiac defibrillator or pacemaker, cardiac ablation, TAVI MI, or PCI within the past year	N= 243, 0.8% women Overall: 68.0 ± NR (45–92) years Frail: 71 ± NR (49–92) years	Hypertension (80.7%), hyperlipidemia (77.0%), CAD (81.5%), PCI (39.5%), MI (30%), PAD (15.2%), depression (23.0%), anxiety (2.1%), and tobacco use (22.6%) at admission
MacEachern [40] 2021 Canada	To examine whether patient frailty levels affect their goal attainment in CR.	Prospective cohort study 1 centre	Patients with heart disease	N = 759, 27.0% women 59.5 ± 9.8 (range NR) years	NR
MacEachern [48] 2023 Canada	To compare the changes in frailty levels from CR admission to completion in patients who enrolled in either centre-based CR or virtual-based CR, and to determine if admission frailty affects frailty changes and cardiovascular risk factors in both programme models.	Retrospective observational study 1 centre	Patients referred to CR following an acute cardiovascular event by an automated referral system or a healthcare professional (e.g., a cardiologist)	N = 132, 36.4% women 64.5 ± 10.5 (40–90) years	Dyslipidemia (89.4%), hypertension (77.3%), diabetes mellitus (28.8%), current smoker (14.4%), CAD (18.9%), ACS (10.6%), MI (47.0%), CABG (15.9%), cardiomyopathy (3.8%), PCI (38.6%) at admission
MacEachern [8] 2024 Canada	To examine if frailty influences achieving goals in CR.	Retrospective observational study 1 centre	Patients referred to CR	N = 759, 23.6% women 59.5 ± 9.8 (range NR) years	CAD (30.6%), MI (33.2%), PCI (7.8%), surgery (19.2%), HF (7.5%), other (1.7%) at admission
Mathew [28] 2019 Canada	To determine if frailty improves with CR and to assess the impact of frailty on CR completion rates.	Retrospective cohort study 1 centre	Patients referred to CR	N = 764, 26.8% women Overall: 64.5 ± 11.9 (range NR) years	Dyslipidemia (0.0%), diabetes mellitus (30.0%), hypertension (70.8%), atrial fibrillation (18.6%) at admission
Mudge [49] 2021 Australia	To describe the characteristics, exercise participation, and outcomes of frail and non-frail participants enrolled in a randomized trial of exercise training within a CR programme.	Retrospective observational study Multicentre (5 centres)	Adults hospitalized with clinical evidence of acute HF	N = 256, 24.2% women NR	Race: 218 (85.2%) Caucasian, 38 (14.8%) other
Nagatomi [53] 2022 Japan	To investigate the efficacy and safety of a comprehensive home-based CR programme using information and communication technology.	Randomized control trial 1 centre	Outpatients with chronic HF and physical frailty	N = 30, 46.7% women 63.7 ± 10.1(range NR) years	Hypertension (20.0%), diabetes mellitus (17.0%), hyperlipidemia (20.0%), CKD (23.0%), PCI (10.0%), valvular surgery (10.0%), PMI (10.0%), ICD (20.0%) at admission
Nelson [50] 2022 USA	To examine the relationship between adherence to the REHAB-HF intervention and trial outcomes, as well as to identify baseline factors associated with adherence.	Retrospective observational study Multicentre (7 clinical sites)	Older acute HF patients	N = 175, 49% women 73.1 ± 8.5 (range NR)years	Hypertension (91.0%), atrial fibrillation (51.0%), hyperlipidemia (63.0%), diabetes mellitus (59.0%), smoking (10.0%), more than high school education (80.0%) at admission
Nishitani-Yokoyama [46] 2021 Japan	To investigate the complaints and prevalence of constipation in patients undergoing CR and the association between constipation and frailty components.	Cross-sectional study 1 centre	Patients with CVD (CAD, open-heart surgery, HF, aorta disease, macrovascular surgery, stenting, PAD and TAVI.	N = 102, 33.3% women 62.7 ± 13.4 (range NR) years	Hypertension (58.0%), diabetes mellitus (12.0%), dyslipidemia (29.0%), current smoker (18.0%), HF (63.0%), aortic disease (21.0%), MI (16.0%), atrial fibrillation (25.0%) at admission
Quach [51] 2023 Canada	To [1] examine the association between frailty and long-term outcomes and to [2] Investigate the association between frailty changes during CR and long-term outcomes.	Retrospective observational study 1 centre	CR participants	N = 3371, 25.7% women 61.9 ± 10.7 (21–94) years	CAD (26.7%), MI (28.3%), HF (6.6%), current smoker (10.9%), education: (31.8%) technical college, marital status: (76.6%) married/living with a partner at admission
Rogers [54] 2018 UK	To inform the feasibility and design of future randomized control trials.	Randomized control trial (pilot) 1 centre	Patients scheduled for TAVI	N = 27, 55.6% women 82.04 ± 4.8 (range NR) years	Diabetes mellitus (14.8%), smoking (48.1%) at admission
Tarro Genta [41] 2015 Italy	To compare the safety and outcome of residential CR in octogenarians after TAVI or AVR.	Prospective cohort study Multicentre [2]	TAVI and sAVR patients, aged ≥80 years participating in CR	N = 110, 64.5% women Overall: 84.0 ± 2.0 (range NR) years	NR
Toshie Tanaka [29] 2018 Japan	To assess the effects of outpatient CR on elderly patients, especially in frailty status.	Retrospective cohort study NR	Patients hospitalized with CVD	N = 47, % women NR Age: NR	NR
Ushijima [42] 2021 Japan	To investigate the effect of CR on the physical function as well as exercise capacity in elderly CVD patients with frailty.	Prospective cohort study 1 centre	CVD patients	N = 89, 23.6% women 75.0 ± 6.0 (range NR) years	Hypertension (62.9%), diabetes mellitus (29.2%), dyslipidemia (66.3%), smoking (13.5%), MI (16.9%), HF (9.0%), aortic disease (11.1%) at admission
Xu [47] 2023 Japan	To investigate the relationship between frailty and health-related quality of life in elderly patients undergoing CR.	Cross-sectional study 1 centre	Elderly patients undergoing CR	N = 217, 33.0% women 74.6 ± 5.8 (range NR) years	NR
Yokote [30] 2023 Japan	To assess whether patients undergoing outpatient CR who have frailty and depressive symptoms at discharge are less likely than those without these condition to establish positive exercise habits.	Retrospective cohort study 1 centre	CR participants	N = 242, 28.7% women 68.2 ± 11.1 (range NR) years	HF (23.4%), IHD (46.7%), depressive symptoms only (8.7%), frailty and depressive symptoms (4.1%) at admission
Yu [31] 2021 China	To assess the changes In CGA, including self-care ability, cognitive function, nutritional status, anxiety, depression and frailty index, and exercise capacity in such CR strategy; to explore the associated factors of the change in exercise capacity.	Retrospective cohort study 1 centre	Patients scheduled for TAVI	N = 90, 40.0% women 74.7 ± 8.1 (range NR) years	Hypertension (71.0%), hyperlipidemia (78.0%), diabetes mellitus (30.0%), smoking (33.0%), CHD (51.0%), PCI (20.0%), CABG (4.0%), MI (6.0%), PAD (26.0%) at admission

^‡^ Includes cardiovascular disease diagnoses and comorbidities as documented in patient medical history, cardiac rehabilitation referral forms, or during programme admission. This information reflects conditions reported or identified prior to or at the time of enrollment in the CR programme. Abbreviations: ACS = acute coronary syndrome; ADHF = acute decompensated heart failure, AVI = aortic valve insufficiency; CABG = coronary artery bypass graft; CAD = coronary artery disease; CGA = comprehensive geriatric assessment; CFS = clinical frailty scale; CHD = congenital heart disease; CKD = chronic kidney disease; COPD = chronic obstructive pulmonary disease; CR = cardiac rehabilitation; CRF = chronic renal failure; CVD = cardiovascular disease; DCM = dilated cardiomyopathy; HCM = hypertrophic cardiomyopathy; HF = heart failure; ICD = implantable cardioverter defibrillator; IHD = ischemic heart disease; MI = myocardial infarction; NR = not reported; NSTEMI = Non-ST-Elevation Myocardial Infarction; PAD = peripheral artery disease; PCI = percutaneous coronary intervention; RCM = restrictive cardiomyopathy; sAVR = surgical aortic valve replacement; STEMI = ST-segment elevation myocardial infarction; TAVI = transcatheter aortic valve implantation; VHD = valvular heart disease.

**Table 2:** Characteristics of the CR programmes in the included studies (N = 39).

First Author	Location	Mode	Duration	Frequency	Components
Adachi [32]	Hospital and Rehab Centre	Centre-based	150 days	3×/week	Exercise training, patient education
Baldasseroni [33]	Rehab Centre	Centre-based	4 weeks	5×/week	Exercise training (biking or callisthenics, 30 min/session)
Bauer [20]	NR	NR	NR	NR	NR
Bencivenga [34]	Rehab Centre	Centre-based	NR	NR	Exercise training, lifestyle modification, psychological support
Dinesh [18]	NR	Hybrid	NR	NR	Exercise training
Eichler [35]	NR	NR	NR	NR	NR
Fonteles Ritt [21]	NR	NR	NR	NR	NR
Francesco Cacciatore [17]	NR	NR	NR	NR	NR
German-Sallo [19]	Rehab Centre	NR	NR	NR	NR
Gilbert [52]	Hospital and Rehab Centre	Centre-based	12 weeks	3×/week	Exercise training
Hashimoto [36]	Rehab Centre	Centre-based	4 months	1×/week	Exercise training
Henderson [37]	NR	Centre-based	≥24 sessions	NR	Exercise training, risk factor education
Hillier [22]	Community centre	Centre-based	12 weeks	2×/week exercise, 1×/week education	Exercise training, patient education
Honzawa [38]	Hospital	Centre-based	150 days	1–2×/week	Risk stratification, exercise training, patient education, psychosocial support
Honzawa [43]	Hospital	Centre-based	NR	NR	Medical evaluation, exercise training, patient education, psychosocial support
Iritani [44]	Hospital	NR	NR	NR	NR
Kamiya [23]	Hospital	Centre-based	5 months	3–5×/week	Exercise training, patient education
Kehler [39]	Community centre	Centre-based	12 weeks	2×/week exercise, 1×/week education	Exercise training, patient education
Kimber [24]	NR	Centre-based	16 weeks	NR	Exercise training, patient education
Kunimoto [45]	Hospital	NR	NR	NR	Medical evaluation, exercise training, patient education, psychosocial support
Landry [25]	NR	NR	24 weeks	NR	Women’s only programme
Lutz [26]	NR	Centre-based	NR	NR	NR
Lutz [27]	Rehab Centre	Centre-based	2–6 weeks	2–3×/week	Exercise training, medication reconciliation, patient education
MacEachern [40]	NR	Centre-based	12 weeks	2×/week exercise, 1×/week education	Exercise training, patient education
MacEachern [48]	NR	Hybrid	Supervised: 6 weeks; Non-supervised: 10 weeks	Supervised: 1×/week exercise, up to 3×/week education; Non-supervised: 150 min/week exercise, up to 4 group sessions/week + up to 6 individualized sessions/week education	Exercise training, patient education
MacEachern [8]	Hospital	Centre-based	12 weeks	Up to 2×/week exercise, 1×/week education	Exercise training, patient education
Mathew [28]	NR	NR	12 weeks	NR	NR
Mudge [49]	Hospital and Home	Hybrid	6 months	2×/week first 3 months, 1×/week subsequent 3 months exercise, 1×/week education	Exercise training, patient education, telephone and clinic follow-up, and medication titration
Nagatomi [53]	NR	Home-based	3 months	Up to 3–5×/week aerobic exercise + 2–3×/week resistance training, education NR	Exercise training, patient education, self-management, nutrition guidance
Nelson [50]	Rehab Centre and Home	Hybrid	12 weeks	3×/week	Exercise training
Nishitani-Yokoyama [46]	NR	NR	NR	NR	NR
Quach [51]	NR	Centre-based	12 weeks	2×/week exercise, 1×/week education	Exercise training, patient education, risk stratification, nutrition guidance, psychosocial support
Rogers [54]	NR	Centre-based	6 weeks	1×/week	Exercise training, patient education
Tarro Genta [41]	NR	NR	3 weeks	2×/day	Exercise training
Toshie Tanaka [29]	Rehab Centre	Centre-based	NR	NR	NR
Ushijima [42]	Rehab Centre	Centre-based	3 months	3–5×/week	Exercise training, patient education, nutrition guidance, medication guidance.
Xu [47]	Hospital	Centre-based	NR	NR	NR
Yokote [30]	Hospital	Centre-based	3 months	1×/week	Exercise training, patient education
Yu [31]	NR	Home-based	NR	5–6×/week	Exercise training

Legend: NR = Not Reported.

**Table 3:** Characteristics of frailty assessment tools and frailty outcomes in CR programmes.

Frailty Assessment Tool(s) Used	Tool Description	First Author Year Country	Time of Assessment	Pre-CR Frailty Prevalence	Post-CR Frailty Prevalence
Kihon Checklist (KCL)	25 yes/no items across 7 domains (activities of daily living, physical function, nutritional status, oral function, social activities of daily living, cognitive function, depressive mood of participants). ≥8 = frail, 4–7 = prefrail, ≤3 = non-frail.	Honzawa [38] 2022 Japan	Pre-CR	Frail: n = 34 (24.8%)	NR
		Honzawa [43] 2020 Japan	Pre-CR	Frail: n = 99 (38.8%)	NR
		Kunimoto [45] 2019 Japan	Pre-CR	Frail: n = 288 (34.1%)	NR
		Nagatomi [53] 2022 Japan	Pre-CR	Frail: n = 10 (33.0%)	NR
		Nishitani-Yokoyama [46] 2021 Japan	Pre-CR	Frail: n = 35 (34.0%)	NR
		Xu [47] 2023 Japan	Pre-CR	Frail: n = 81 (37.0%)	NR
Fried’s Frailty Criteria	Assesses weight loss, exhaustion, grip strength, walking speed, and physical activity. Frailty if ≥3 criteria met.	Francesco-Cacciatore [17] 2013 Italy	Pre-CR	Frail: n = 109 (31.1%)	NR
		Gilbert [52] 2023 USA	Post-CR	NR	NR
		Nelson [50] 2022 USA	Pre-CR	Frail: n = 92 (53.0%)	NR
		Rogers [54] 2018 UK	Pre-CR and Post-CR	Frailty by FRIED scale: 3, n = 5/25 (20.0%), 2, n = 8/25 (32.0%), 1, n = 9/25 (36.0%)	Frailty by FRIED scale: 3, n = 1/14 (7.1%), 2, n = 6/14 (42.9%), 1, n = 6/14 (42.9%)
		Toshie Tanaka [29] 2018 Japan	Pre-CR and Post-CR	Frail: n = 8 (25.8%)	Frail: n = 2 (6.5%)
		Yu [31] 2021 China	Pre-CR and Post-CR	Frail: n = 75 (83.0%)	Frail: n = 23 (26.0%)
25-Item Frailty Index (FI)	Domains: (1) CV risk factors, (2) CV symptoms (NYHA class), (3) stress test, (4) Quality of life (SF-36), (5) body composition, (6) diet. Score: 0–1. Categorized by 0.1 increments.	Hillier [22] 2023 Canada	Pre-CR	Frailty by FI categories: >0.5 FI: n = 227 (9.0%), 0.4–0.5 FI: n = 462 (17.0%), 0.3–0.4 FI: n = 791 (30.0%), 0.2–0.3 FI: n = 715 (27.0%), <0.2 FI: n = 448 (17.0%)	NR
		Kehler [39] 2020 Canada	Pre-CR and Post-CR	Frailty by FI categories: >0.50 FI: 175 (7.5%), 0.4–0.5 FI: n = 401 (17.3%), 0.3–0.4 FI: n = 690 (29.7%), 0.2–0.3 FI: n = 642 (27.6%), <0.2 FI: n = 414 (17.8%)	Frailty by FI categories: >0.50 FI: 95 (4.1%), 0.4–0.5 FI: n = 209 (9.0%), 0.3–0.4 FI: n = 447 (19.3%), 0.2–0.3 FI: n = 659 (28.4%), <0.2 FI: n = 912 (39.3%)
		MacEachern [40] 2021 Canada	Pre-CR and Post-CR	NR	NR
		MacEachern [8] 2024 Canada	Pre-CR	Frailty by FI categories: >0.40 FI: n = 204 (26.9%), 0.30–0.39 FI: n = 219 (28.9%), 0.20–0.29 FI: n = 207 (27.3%), <0.2 FI: n = 129 (17.0%)	NR
		Quach [51] 2023 Canada	Pre-CR	Frailty by FI categories: 0.5 > FI: n = 356 (10.6%), 0.4–0.5 FI: n = 650 (19.3%), 0.3–0.4 FI: n = 964 (28.6%), 0.2–0.3 FI: n = 872 (25.9%), <0.2 FI: n = 529 (15.7%)	NR
6-Minute Walk Distance (6MWD)	<300 m = frailty	Henderson [37] 2017 USA	Pre-CR and Post-CR	Frail: n = 24 (40.0%)	Frail: n = 11 (18.3%)
		Lutz [26] 2019 USA	Pre-CR	Frail: n = 49 (30.1%)	NR
		Lutz [27] 2020 USA	Pre-CR	Frail: n = 75 (30.9%)	NR
		Tarro Genta [41] 2015 Italy	Post-CR	NR	Frail: n = 22 (20.0%)
Clinical Frailty Scale (CFS)—also known as Rockwood Frailty Scale	1–9 scale; score ≥ 4 indicates frailty.	Francesco-Cacciatore [17] 2013 Italy	Pre-CR	Frail: n = 90 (25.6%)	NR
		German-Sallo [19] 2024 Hungary	Pre-CR	Frail: n = 30 (32.6%)	NR
		Kimber [24] 2018 Canada	Pre-CR and Post-CR	NR	NR
Edmonton Frail Scale (EFS)	Assesses 10 domains including cognition, nutrition, mood, and function. Score range: 0–17 (higher = more frail).	Fonteles Ritt [21] 2021 Brazil	Pre-CR and Post-CR (≥3 months after start)	Mean EFS: 5.4 ± 2.0 (frailty level not reported as n/%)	Mean EFS score 4.8 ± 1.9 (frailty level not reported as n/%)
		Mathew [28] 2019 Canada	Pre-CR and Post-CR	NR	No improvement in EFS score: n = 489, mean EFS score: 3.2, Any improvement: n = 275, mean EFS score: 5.0, Total: n = 764 (completers), mean EFS score: 3.8
		Rogers [54] 2018 UK	Pre-CR and Post-CR	EFS: 5.08 (2.2)	EFS: 4.4 (1.7)
Gait Speed (GS)	GS < 1 m/s = frailty	Henderson [37] 2017 USA	Pre-CR and Post-CR	Frail: n = 24 (40.0%)	Frail: n = 11 (18.3%)
		Lutz [26] 2019 USA	Pre-CR	Frail: n = 49 (30.1%)	NR
		Lutz [27] 2020 USA	Pre-CR	Frail: n = 75 (30.9%)	NR
Japanese Version of the Cardiovascular Health Study Standard (J-CHS)—A revised version of Fried’s Frailty Criteria	Assesses 5 domains: weight loss, low activity, fatigue, weakness, gait speed. Frailty if ≥3 criteria present.	Hashimoto [36] 2022 Japan	Pre-CR	Frail: n = 15 (28.8%)	NR
		Ushijima [42] 2021 Japan	Pre-CR and Post-CR	Frail: n = 23 (25.8%)	Frail: n = 3 (3.4%)
		Yokote [30] 2023 Japan	Pre-CR	Frail: n = 48 (19.8%)	NR
Tandem Stand (TS)	TS < 10 s = frailty	Henderson [37] 2017 USA	Pre-CR and Post-CR	Frail: n = 24 (40.0%)	Frail: n = 11 (18.3%)
		Lutz [26] 2019 USA	Pre-CR	Frail: n = 49 (30.1%)	NR
		Lutz [27] 2020 USA	Pre-CR	Frail: n = 75 (30.9%)	NR
Timed Up and Go (TUG)	TUG > 15 s = frailty	Henderson [37] 2017 USA	Pre-CR and Post-CR	Frail: n = 24 (40.0%)	Frail: n = 11 (18.3%)
		Lutz [26] 2019 USA	Pre-CR	Frail: n = 49 (30.1%)	NR
		Lutz [27] 2020 USA	Pre-CR	Frail: n = 75 (30.9%)	NR
FLAGSHIP Frailty Score	Assesses physical frailty in heart failure prognosis. 4 domains: slowness, weakness, inactivity, exhaustion. Score 0–14; higher = worse frailty	Adachi [32] 2023 Japan	Pre-CR	Frail: n = 1062 (39.4%)	NR
		Iritani [44] 2023 Japan	Pre-CR	Frail: n = 1993 (100%)	NR
Hand Grip Strength (HGS)	HGS based on Fried	Lutz [26] 2019 USA	Pre-CR	Frail: n = 49 (30.1%)	NR
		Lutz [27] 2020 USA	Pre-CR	Frail: n = 75 (30.9%)	NR
Modified Fried Criteria (MFC)—A revised version of Fried’s Frailty Criteria	Assesses 5 domains: exhaustion, grip strength, mobility, unintentional weight loss, and physical activity. Score 0: robust; 1–2: prefrail; 3–5: frail.	Dinesh [18] 2024 Australia	Pre-CR and Post-CR (cross-sectional)	Frail: n = 61 (21.3%)	Lower frailty scores in CR groups compared to non-CR groups, n (%) NR
		Kimber [24] 2018 Canada	Pre-CR and Post-CR	NR	NR
Short Physical Performance Battery (SPPB)	Gait speed, balance, chair stand; total score 0–12; <7 = frailty	Baldasseroni [33] 2023 Italy	Pre-CR and Post-CR	Mild frailty: n = 27 (27.0%), moderate frailty: n = 14 (14.0%), severe frailty: n = 0 (0.0%)	Mild frailty: n = 22 (22.0%), moderate frailty: n = 3 (3.0%), severe frailty: n = 0 (0.0%)
		Kimber [24] 2018 Canada	Pre-CR and Post-CR	NR	NR
Barthel Index (BI)	BI < 75	Tarro Genta [41] 2015 Italy	Post-CR	NR	Frail: n = 22 (20.0%)
Claims-Based Frailty Index (Quartiles)	Patients were stratified into quartiles: Q1 = least frail) to Q4 = most frail.	Bauer [20] 2023 USA	Pre-CR	Most frail (Q4 quartile): n = 117,595 (20.6%)	NR
Comprehensive Geriatric Assessment based frailty index (CGA-based FI)	Assesses 40 multidimensional health deficits [56]. Each deficit scored 1 if present, 0 if absence. FI = total score/number of items. Cut-off ≥ 0.25 defines frailty.	Bencivenga [34] 2022 Italy	Pre-CR	Frail: n = 293 (52.4%)	NR
FRAIL Scale (FS)	Assesses fatigue, resistance, ambulation, illnesses, weight loss; score 0–5. Frail if FS ≥ 3 or CFS ≥ 5.	German-Sallo [19] 2024 Hungary	Pre-CR	Frail: n = 30 (32.6%)	NR
Functional Frailty Index (FFI)	FFI based on 25 items across domains; score ≥ 0.25 = frailty.	Kimber [24] 2018 Canada	Pre-CR and Post-CR	NR	NR
Lachs Frailty Scale	Based on absence of comorbidity.	Francesco-Cacciatore [17] 2013 Italy	Pre-CR	Frail: n = 138 (39.3%)	NR
Morse Fall Scale (MFS)	MFS ≥ 50	Tarro Genta [41] 2015 Italy	Post-CR	NR	Frail: n = 22 (20.0%)
Multidimensional Geriatric Assessment (MGA)	Scored 0–7; score ≥ 3 indicates probable frailty.	Eichler [35] 2020 Germany	Pre-CR	Frail: n = 76 (61.3%)	NR
Validated Frailty Index Scale	Derived from medical intake history. Classification not described in detail.	Landry [25] 2018 Canada	Pre-CR	Mild frailty: n = 367 (46.0%), moderate frailty: n = 33 (4.1%)	NR
19-Item Frailty Index (FI)	Items scored 0–1. Score = positive items/19. Quartiles define frailty: 0–0.12 = fit, >0.12–0.24 = mild, >0.24–0.36 = moderate, >0.36 = severe.	Kamiya [23] 2020 Japan	Pre-CR	FI score: 0.28 ± 0.13 (frailty level NR as n/%)	NR
41-Item Frailty Index (FI)	Assesses function, mood, comorbidity, self-rated health, nutrition, and cognition. Items scored from 0 (non-frail) to 1 (most frail). FI = sum of scores/number of items. Frailty categorized as: <0.20 = non-frail, 0.20–0.39 = frail, ≥0.40 = very frail.	Mudge [49] 2021 Australia	Pre-CR and Post-CR	Frail: n = 119 (46.0%), very frail: n = 27 (11.0%)	Frail/very frail with improvements at 6 months: n = 87 (34.0%)
65-Item Frailty Index (FI)	Includes symptoms, diseases, disability, and signs. Items scored 0 (non-frail) to 1 (most frail). FI = number of deficits/65. Categories: <0.10 (least frail), 0.11–0.19, 0.20–0.29, >0.30 (most frail).	MacEachern [48] 2023 Canada	Pre-CR	Frailty by FI categories: Centre based; >0.30 FI: n = 2 (2.7%), 0.20–0.29 FI: n = 5 (6.7%), 0.11–0.19 FI: n = 32 (43.2%), <0.10 FI: n = 35 (47.2%) Virtual; >0.30 FI: n = 1 (1.7%), 0.20–0.29 FI: n = 4 (6.8%), 0.11–0.19 FI: n = 24 (41.3%), <0.10 FI: n = 29 (50%)	NR

Legend: CR = Cardiac Rehabilitation; NR = Not Reported; NYHA = New York Heart Association.

**Table 4:** Reported Barriers to CR Participation in Patients with Frailty (n = 8).

Barrier	Description	Study
Physical Limitations	Declined physical capacity; need for modifications addressing strength, balance, and frailty-specific hazards	Iritani [44], Henderson [37]
Mental Health and Emotional Factors	Anxiety, depression, emotional distress, and role-emotional limitations related to frailty	Hillier [22], Honzawa [38], Honzawa [43]
Motivational Barriers	Lack of motivation	Iritani [44]
Transportation and Access	No transportation	Iritani [44], Rogers [54]
Healthcare System-Related Factors	Medical appointments and coordination issues with multiple healthcare providers	Nelson [50]
Personal and Social Factors	Conflicting personal commitments (e.g., work, childcare, travel), lack of social support, or caregiver responsibilities.	Nelson [50]
Cognitive Impairment	Need for CR modifications to support cognition	Henderson [37]
Low Baseline Physical Activity	Frailer participants are significantly less likely to meet PA guidelines	Mudge [49]

Abbreviations: CR = cardiac rehabilitation, PA = physical activity.

## Data Availability

Data is available upon request.

## Supplementary Materials

The following supporting information can be downloaded at: https://www.mdpi.com/article/10.3390/jcm14155354/s1, File S1: Search Strategy.
