# Supplementary material for: Understanding Frailty in Cardiac Rehabilitation: A Scoping Review of Prevalence, Measurement, Sex and Gender Considerations, and Barriers to Completion
Source: J Clin Med. 2025 Jul 29;14(15):5354. doi: 10.3390/jcm14155354 (PMC12347422; doi:10.3390/jcm14155354)
Supplement: Supplementary file 1 [file jcm-14-05354-s001.zip › jcm-3774374-supplementary.pdf]

Supplementary File S1

Medline

Database: Ovid MEDLINE(R) ALL <1946 to May 14, 2025>

#	Query	Results from 15 May 2025
1	Cardiac Rehabilitation/ or exp Cardiovascular Diseases/rh	20,559
2	((cardi* or heart* or vascul* or myocardi* or coronary) adj3 (rehab* or telerehab* or prehab* or secondary prevent*)).ti,ab,kf,jw.	19,350
3	or/1-2 [Cardiac Rehab]	31,940
4	Frailty/ or Frail Elderly/	24,797
5	Geriatric Assessment/	35,154
6	(frail* or defrail* or debility or debilities or fragil* or infirm* or function* impair*).ti,ab,kf.	146,603
7	(comprehensive geriatr* assessment* or Kihon Checklist*).ti,ab,kf.	3,985
8	((elder* or aged or ag?ing or old or older or senior* or geriatr* or sexagen* or septuagen* or octogen* or nonagen* or centenar*) adj3 (vulnerab* or multimorbid* or multi*-morbid* or multi*-chronic*)).ti,ab,kf.	7,830
9	((elder* or aged or ag?ing or old or older or senior* or geriatr* or sexagen* or septuagen* or octogen* or nonagen* or centenar*) adj3 (assess* or evaluat* or exam*)).ti,ab,kf.	49,060
10	or/4-9 [Frailty]	221,893
**11**	**3 and 10**	**676**

Embase

Database: Embase Classic+Embase <1947 to 2025 May 14>

#	Query	Results from 15 May 2025
1	heart rehabilitation/ or exp cardiovascular disease/rh	45,353
2	((cardi* or heart* or vascul* or myocardi* or coronary) adj3 (rehab* or telerehab* or prehab* or secondary prevent*)).ti,ab,kf,jx.	32,235
3	or/1-2 [Cardiac Rehab]	60,471
4	frailty/ or frail elderly/	48,316
5	geriatric assessment/ or clinical frailty scale/ or edmonton frail scale/ or tilburg frailty indicator/	26,166
6	geriatric rehabilitation/	1,523
7	(frail* or defrail* or debility or debilities or fragil* or infirm* or function* impair*).ti,ab,kf.	210,161
8	(comprehensive geriatr* assessment* or Kihon Checklist*).ti,ab,kf.	7,070
9	((elder* or aged or ag?ing or old or older or senior* or geriatr* or sexagen* or septuagen* or octogen* or nonagen* or centenar*) adj3 (vulnerab* or multimorbid* or multi*-morbid* or multi*-chronic*)).ti,ab,kf.	10,212
10	((elder* or aged or ag?ing or old or older or senior* or geriatr* or sexagen* or septuagen* or octogen* or nonagen* or centenar*) adj3 (assess* or evaluat* or exam*)).ti,ab,kf.	72,325
11	or/4-10 [Frailty]	299,761
**12**	**3 and 11**	**1,330**

CENTRAL

Database: EBM Reviews - Cochrane Central Register of Controlled Trials <April 2025>

#	Query	Results from 15 May 2025
1	Cardiac Rehabilitation/	631
2	((cardi* or heart* or vascul* or myocardi* or coronary) adj3 (rehab* or telerehab* or prehab* or secondary prevent*)).ti,ab,kw,jw.	5,938
3	or/1-2 [Cardiac Rehab]	5,996
4	Frailty/ or Frail Elderly/	1,837
5	Geriatric Assessment/	2,046
6	(frail* or defrail* or debility or debilities or fragil* or infirm* or function* impair*).ti,ab,kw.	13,844
7	(comprehensive geriatr* assessment* or Kihon Checklist*).ti,ab,kw.	567
8	((elder* or aged or ag?ing or old or older or senior* or geriatr* or sexagen* or septuagen* or octogen* or nonagen* or centenar*) adj3 (vulnerab* or multimorbid* or multi*-morbid* or multi*-chronic*)).ti,ab,kw.	900
9	((elder* or aged or ag?ing or old or older or senior* or geriatr* or sexagen* or septuagen* or octogen* or nonagen* or centenar*) adj3 (assess* or evaluat* or exam*)).ti,ab,kw.	9,810
10	or/4-9 [Frailty]	24,938
**11**	**3 and 10**	**202**

Emcare

Database: Ovid Emcare Nursing <1995 to Present>

#	Query	Results from 15 May 2025
1	heart rehabilitation/ or exp cardiovascular disease/rh	10,414
2	((cardi* or heart* or vascul* or myocardi* or coronary) adj3 (rehab* or telerehab* or prehab* or secondary prevent*)).ti,ab,kf,jx.	9,413
3	or/1-2 [Cardiac Rehab]	14,413
4	frailty/ or frail elderly/	20,565
5	geriatric assessment/ or clinical frailty scale/ or edmonton frail scale/ or tilburg frailty indicator/	8,195
6	geriatric rehabilitation/	1,487
7	(frail* or defrail* or debility or debilities or fragil* or infirm* or function* impair*).ti,ab,kf.	60,646
8	(comprehensive geriatr* assessment* or Kihon Checklist*).ti,ab,kf.	2,754
9	((elder* or aged or ag?ing or old or older or senior* or geriatr* or sexagen* or septuagen* or octogen* or nonagen* or centenar*) adj3 (vulnerab* or multimorbid* or multi*-morbid* or multi*-chronic*)).ti,ab,kf.	5,078
10	((elder* or aged or ag?ing or old or older or senior* or geriatr* or sexagen* or septuagen* or octogen* or nonagen* or centenar*) adj3 (assess* or evaluat* or exam*)).ti,ab,kf.	27,347
11	or/4-10 [Frailty]	94,799
**12**	**3 and 11**	**344**

CINAHL

Expanders - Apply equivalent subjects
Search modes - Proximity
Interface - EBSCOhost Research Databases
Search Screen - Advanced Search

Database: CINAHL Ultimate

#	Query	Results from 15 May 2025
S1	(MH "Rehabilitation, Cardiac+") OR (MH "Cardiovascular Diseases+/RH")	22,352
S2	TI ((cardi* or heart* or vascul* or myocardi* or coronary) N2 (rehab* or telerehab* or prehab* or secondary prevent*)) OR<br>AB ((cardi* or heart* or vascul* or myocardi* or coronary) N2 (rehab* or telerehab* or prehab* or secondary prevent*)) OR<br>SO ((cardi* or heart* or vascul* or myocardi* or coronary) N2 (rehab* or telerehab* or prehab* or secondary prevent*))	8,502
S3	S1 OR S2	26,645
S4	(MH "Frailty Syndrome") OR (MH "Frail Elderly")	14,706
S5	(MH "Geriatric Assessment+")	19,637
S6	(MH "Rehabilitation, Geriatric")	3,288
S7	TI (frail* or defrail* or debility or debilities or fragil* or infirm* or function* impair*) OR<br>AB (frail* or defrail* or debility or debilities or fragil* or infirm* or function* impair*)	44,577
S8	TI (comprehensive geriatr* assessment* or Kihon Checklist*) OR<br>AB (comprehensive geriatr* assessment* or Kihon Checklist*)	1,833
S9	TI ((elder* or aged or ag#ing or old or older or senior* or geriatr* or sexagen* or septuagen* or octogen* or nonagen* or centenar*) N2 (vulnerab* or multimorbid* or multi*-morbid* or multi*-chronic*)) OR<br>AB ((elder* or aged or ag#ing or old or older or senior* or geriatr* or sexagen* or septuagen* or octogen* or nonagen* or centenar*) N2 (vulnerab* or multimorbid* or multi*-morbid* or multi*-chronic*))	3,925
S10	TI ((elder* or aged or ag#ing or old or older or senior* or geriatr* or sexagen* or septuagen* or octogen* or nonagen* or centenar*) N2 (assess* or evaluat* or exam*)) OR<br>AB ((elder* or aged or ag#ing or old or older or senior* or geriatr* or sexagen* or septuagen* or octogen* or nonagen* or centenar*) N2 (assess* or evaluat* or exam*))	19,386
S11	S4 OR S5 OR S6 OR S7 OR S8 OR S9 OR S10	84,645
**S12**	**S3 AND S11**	**608**

Web of Science

Database:
Web of Science Core Collection
Editions: All

- WOS.IC: 1993 to 2025
- WOS.CCR: 1985 to 2025
- WOS.SCI: 1900 to 2025
- WOS.AHCI: 1975 to 2025
- WOS.BHCI: 2005 to 2025
- WOS.BSCI: 2005 to 2025
- WOS.ESCI: 2005 to 2025
- WOS.ISTP: 1990 to 2025
- WOS.SSCI: 1900 to 2025
- WOS.ISSHP: 1990 to 2025

Exact Search = On

#	Query	Results from 15 May 2025
1	TS=((cardi* or heart* or vascul* or myocardi* or coronary) NEAR/2 (rehab* or telerehab* or prehab* or "secondary prevent*")) OR SO=(cardiac rehabilitation or cardiac rehabilitation in women or european journal of cardiovascular prevention rehabilitation or journal of cardiopulmonary rehabilitation "and" prevention)	23,043
2	TS=(frail* or defrail* or debility or debilities or fragil* or infirm* or "function* impair*") OR TS=("comprehensive geriatr* assessment*" or "Kihon Checklist*") OR TS=((elder* or aged or ag$ing or old or older or senior* or geriatr* or sexagen* or septuagen* or octogen* or nonagen* or centenar*) NEAR/2 (vulnerab* or multimorbid* or multi*-morbid* or multi*-chronic*)) OR TS=((elder* or aged or ag$ing or old or older or senior* or geriatr* or sexagen* or septuagen* or octogen* or nonagen* or centenar*) NEAR/2 (assess* or evaluat* or exam*))	293,167
**3**	**#1 AND #2**	**534**
